# Personalized neoantigen DC vaccine combined with camrelizumab following definitive therapy in locally advanced unresectable esophageal squamous cell carcinoma (CHANT-241): protocol for a randomized controlled trial

**DOI:** 10.3389/fimmu.2025.1658670

**Published:** 2026-01-12

**Authors:** Yueyun Chen, Yue Zheng, Huashan Shi, Wenjie Yang, Heng Xu, Yang Li, Zhenyu Ding

**Affiliations:** 1 Department of Biotherapy, Cancer Center and State Key Laboratory of Biotherapy, West China Hospital, Sichuan University, Chengdu, Sichuan, China; 2 Center of Biostatistics, Design, Measurement and Evaluation (CBDME), West China Hospital, Sichuan University, Chengdu, Sichuan, China

**Keywords:** DC vaccine, esophageal squamous cell carcinoma, immunotherapy, neoantigen, personalized cancer therapy

## Abstract

**Importance:**

Locally advanced, unresectable esophageal squamous cell carcinoma (ESCC) has a poor prognosis despite definitive chemoradiotherapy (CRT), and no standard maintenance therapy currently exists. Personalized vaccines targeting tumor neoantigens combined with immune checkpoint inhibitors may enhance antitumor immunity, potentially improving these patients’ outcomes.

**Objective:**

To evaluate whether maintenance therapy with a personalized neoantigen dendritic cell vaccine (Neo-DCVac) combined with camrelizumab improves overall survival (OS) compared to camrelizumab alone in patients with unresectable locally advanced ESCC following definitive CRT.

**Design, setting, and participants:**

The CHANT-241 trial is a randomized, open-label, single-center, phase 2 clinical trial enrolling 165 patients aged 18 to 80 years with histologically confirmed unresectable locally advanced ESCC. Eligible participants must have completed definitive chemoradiotherapy (CRT) and undergone radiologic assessment within 3 to 5 weeks demonstrating no evidence of disease progression. Prior immunotherapy is allowed. Additional inclusion criteria include the ability to provide fresh tumor tissue or archived pathology slides of sufficient quality. Patients are randomized in a 2:1 ratio to receive either combination therapy or camrelizumab alone.

**Intervention:**

Patients in the experimental group receive Neo-DCVac (0.5–2 × 10^7^ cells per dose, subcutaneously, following cyclophosphamide pretreatment), administered as 5 priming doses and 10 booster doses over a 12-month vaccination period, in combination with camrelizumab (200 mg intravenously every 4 weeks). Patients in the control arm receive camrelizumab alone at the same dose and schedule.

**Main outcomes and measures:**

The primary endpoint is the 2-year OS rate. Secondary endpoints include OS, progression-free survival (PFS), treatment-related adverse events (TRAEs), and exploratory biomarker analyses, including tumor mutational burden (TMB), PD-L1 expression, and circulating tumor DNA (ctDNA).

**Results:**

Clinical outcomes are not yet available. Upon completion of enrollment and data analysis, the study findings will be disseminated through publication in a peer-reviewed journal.

**Conclusions:**

The CHANT-241 trial is designed to evaluate whether the addition of Neo-DCVac to camrelizumab as maintenance therapy improves survival outcomes in patients with unresectable locally advanced ESCC. The findings aim to provide high-level evidence supporting a novel precision immunotherapy approach in this population.

**Clinical Trial Registration:**

ClinicalTrials.gov, identifier NCT06675201.

## Introduction

Esophageal squamous cell carcinoma (ESCC) is the predominant histological subtype of esophageal cancer in East Asia, particularly in China, accounting for over 50% of global incidence and mortality (1–3). Due to the absence of early clinical symptoms, most patients with ESCC are diagnosed at locally advanced or metastatic stages. Curative treatment options for these patients are limited, treatment-related toxicity is substantial, and prognosis remains poor (4). Concurrent chemoradiotherapy (CRT) remains the standard treatment for patients with unresectable locally advanced ESCC. However, rapid tumor recurrence or distant metastasis frequently occurs post-CRT, representing a primary cause of treatment failure. Currently, no standard maintenance therapy exists for this patient population after definitive CRT, underscoring the urgent need for effective post-CRT therapeutic strategies.

Recent research has explored immune checkpoint inhibitors (ICIs) as potential maintenance therapies for ESCC. Ongoing phase II and III clinical trials, including ESCORT-CRT, SCORT-CR, RATIONALE-311, KUNLUN, and KEYNOTE-975, are evaluating the efficacy of ICIs in this setting. Collectively, these studies suggest that ICIs could represent a promising maintenance approach following definitive treatment for unresectable locally advanced ESCC.

**Figure 1:**
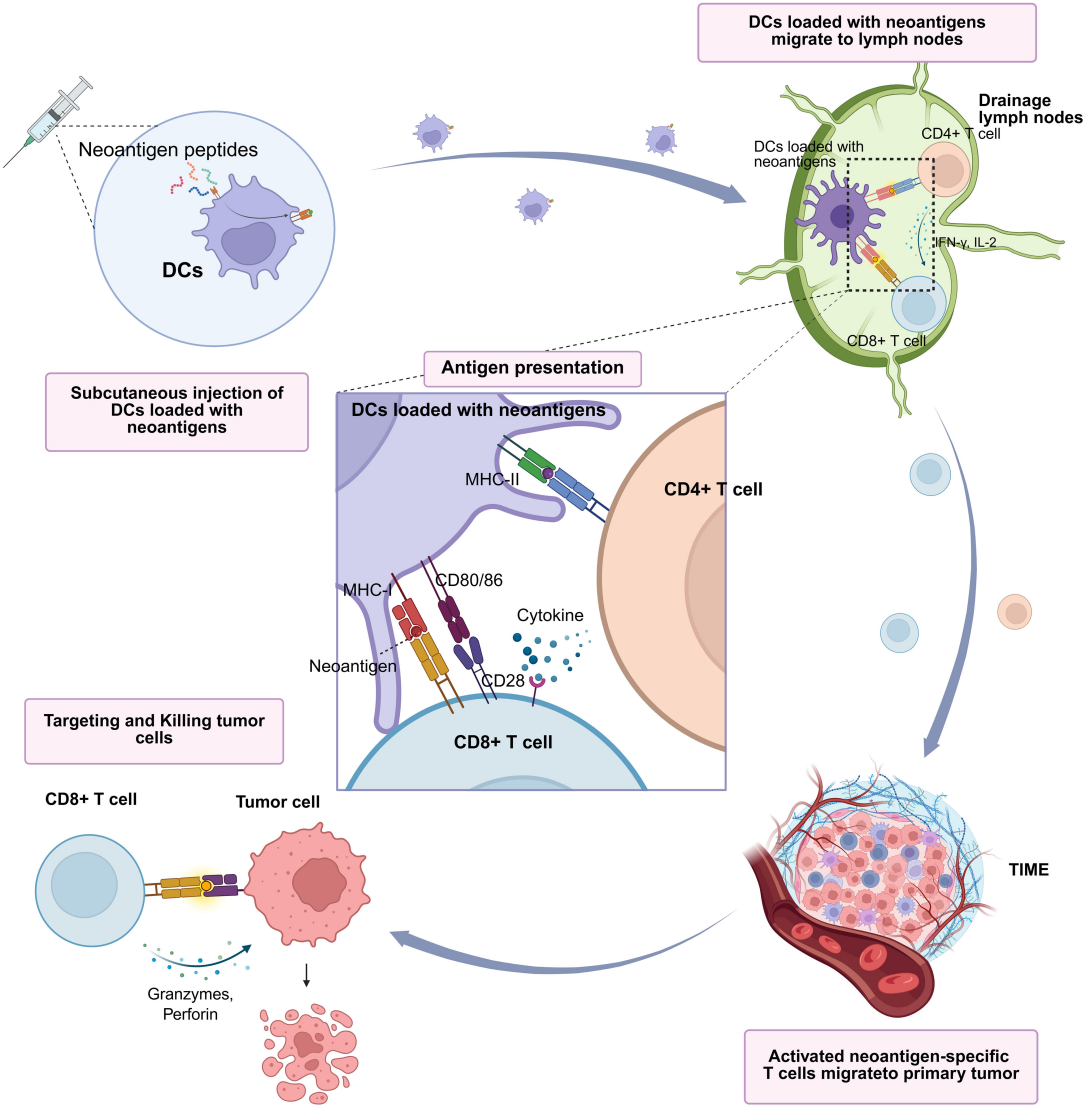
Overview of the immune mechanism of neoantigen-loaded dendritic cell (DC) vaccines. DCs capture and process selected neoantigen peptides, subsequently presenting them via MHC class I and II pathways to elicit antigen-specific T cell responses, ultimately leading to tumor cell elimination. iDCs, immature dendritic cells; IFN-γ, interferon-gamma; IL-12, interleukin-12; MHC, major histocompatibility complex.

Among immunotherapeutic modalities, tumor vaccines have emerged as promising treatments due to their ability to elicit or enhance endogenous antitumor immune responses. Neoantigen-specific T cells, capable of recognizing tumor-specific mutations, are crucial effectors in immune-mediated tumor control, making them ideal targets for personalized cancer vaccines (5, 6). Advances in next-generation sequencing (NGS) technologies have accelerated the development of neoantigen-based vaccines (7–9). Dendritic cell (DC), the primary antigen-presenting cells, initiate adaptive immune responses by presenting tumor-specific antigens, thus promoting robust anti-tumor T cell activity within the tumor microenvironment (10, 11). Numerous clinical studies are evaluating the application of DC-based vaccines across various solid tumors, demonstrating initial safety and preliminary efficacy (12–15). DC vaccines function by presenting tumor antigens to CD8^+^ T cells via MHC class I molecules, thereby enhancing cytotoxic T lymphocyte (CTL) activity. Furthermore, they engage CD4^+^ T cells through MHC class II-mediated antigen presentation, providing necessary co-stimulatory signals and cytokine support for sustained CD8^+^ T cell activation (16–19) (**Figure 1**).

Esophageal cancer treatment has entered an era increasingly dominated by immunotherapy (20–24). Specifically, ESCC is characterized by a high tumor mutational burden (TMB), suggesting a significant presence of neoantigens and thus positioning ESCC as an optimal candidate for neoantigen-based vaccine therapies. Recently, the combination of camrelizumab (Jiangsu Hengrui Pharmaceuticals Co Ltd., China), a humanized anti–programmed death 1 (PD-1) monoclonal antibody, with chemotherapy was approved in China as a first-line treatment for patients with advanced or metastatic ESCC. Clinical trials evaluating this regimen have demonstrated substantial survival advantages alongside manageable safety profiles (20, 25, 26). Emerging evidence further indicates that blockade of the PD-1 and programmed death ligand 1 (PD-L1) pathway can significantly enhance neoantigen-specific T cell responses (27–30). Our preliminary clinical studies in ESCC patients have shown that Neo-DCVac demonstrated an acceptable safety profile and exhibit promising antitumor activity (31). Furthermore, combination therapies integrating Neo-DCVac and ICIs have showed synergistic efficacy in our previous study (14) and compassionate-use scenarios in postoperative adjuvant settings for locally advanced ESCC (CHANT-211 study). Furthermore, ongoing phase 1 trials continue to evaluate the safety and preliminary efficacy of Neo-DCVac administered in combination or sequentially with ICIs in various malignancies, including ICI-resistant lung cancer (NCT05235607), colorectal cancer (CHANT-191 trial, NCT04147078), and pancreatic cancer (CHANT-231 trial, NCT06344156). Based on these compelling preliminary results, we hypothesize that Neo-DCVac maintenance therapy, combined with ICIs post-CRT, could improve overall survival in patients with unresectable locally advanced ESCC. This personalized therapeutic strategy is expected to effectively address the substantial unmet clinical need in this patient population.

## Methods

This study was approved by the Institutional Review Board of West China Hospital, Sichuan University (Approval No. 2024-1563). Written informed consent will be obtained from all participants prior to enrollment. The trial has been registered on ClinicalTrials.gov (NCT06675201) and will be conducted in accordance with the Declaration of Helsinki (64th WMA General Assembly, Fortaleza, Brazil, 2013). Patients and members of the public were not involved in the design, conduct, reporting, or dissemination of this research.

### Study design

This is a prospective, open-label, randomized controlled phase 2 trial designed to evaluate the clinical efficacy of a personalized Neo-DCVac as maintenance therapy in patients with unresectable locally advanced ESCC following definitive chemoradiotherapy. Eligible patients with histologically confirmed ESCC who have completed definitive concurrent or sequential CRT, with or without prior immunotherapy (PD-1/PD-L1), and show no evidence of disease progression at reassessment will be enrolled. Participants will be randomized in a 2:1 ratio to receive either Neo-DCVac plus camrelizumab (experimental group) or camrelizumab monotherapy (control group). Maintenance treatment will continue for up to 12 months or until disease progression, unacceptable toxicity, or any cause of death.

**Figure 2:**
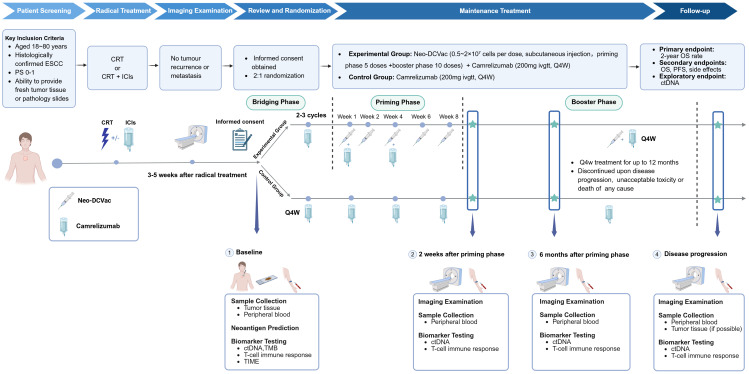
Overview of study design and treatment schedule. Eligible patients are randomized into two groups to receive the assigned interventions. Patients in the experimental group undergo 2–3 cycles of camrelizumab induction therapy while awaiting vaccine preparation. This is followed by prime vaccination administered at weeks 1, 2, 4, 6, and 8, in combination with camrelizumab every 4 weeks. Patients then enter the booster phase, receiving both vaccine and camrelizumab every 4 weeks. In the control group, patients receive camrelizumab monotherapy every 4 weeks. Treatment continues for up to 12 months unless disease progression, unacceptable toxicity, or other termination criteria are met. Radiologic evaluations will be conducted periodically during treatment. Peripheral blood samples will be collected at baseline, 2 weeks and 6 months after the priming phase, and at the time of disease progression for ctDNA and other analyses. Throughout the study, all ongoing treatments and clinical monitoring are conducted under the supervision of oncologists from West China Hospital. CRT, chemoradiotherapy; ICIs, immune checkpoint inhibitors; OS, overall survival; PFS, progress free survival; ctDNA, circulating-tumor DNA; TMB, tumor mutation burden; TIME, tumor immune microenvironment.

At baseline, tumor tissue and peripheral blood samples will be collected from patients for neoantigen screening, circulating tumor DNA (ctDNA) analysis, tumor mutational burden (TMB) assessment, T cell immune response profiling, and evaluation of the tumor immune microenvironment (TIME). Patients will be followed regularly according to standard clinical practice to monitor survival status and late-onset adverse events (AEs). Radiographic assessments will be performed, and peripheral blood samples will be recollected for ctDNA and T cell immune response analysis at 2 weeks and 6 months after the initial Neo-DCVac administration, as well as at the time of disease progression. A schematic of the study design is presented in **Figure 2**.

### Sample size

The sample size was calculated using PASS software (version 15.0; NCSS, LLC, Kaysville, UT, USA; https://www.ncss.com), based on the anticipated difference in the 2-year overall survival (OS) rate. Participants will be randomized in a 2:1 ratio to receive either combination therapy (experimental group) or monotherapy (control group). Assuming a 2-year OS rate of 35% in the control arm and 50% in the experimental arm, with a one-sided significance level (α) set at 0.20, the study is designed to achieve 80% statistical power to detect this clinically meaningful difference (32). A dropout rate of 16% was assumed based on the potential failure of neoantigen prediction. To account for an estimated dropout rate of 16%, a total enrollment of 165 patients is required, including 110 patients in the experimental arm and 55 patients in the control arm.

### Study population

#### Key inclusion criteria

Patients eligible for inclusion must meet all the following criteria:

(1) Male or female patients aged 18 to 80 years; (2) Histologically confirmed locally advanced, unresectable ESCC following definitive therapy (chemoradiotherapy or chemo radioimmunotherapy, restricted to anti–PD-1/PD-L1); (3) No evidence of disease progression according to RECIST 1.1 on CT/MRI performed 3–5 weeks after completion of definitive therapy; (4) Only patients with complete response, partial response, or stable disease are eligible; (5) Availability of fresh tumor tissue samples or archived pathology slides; (6) Eastern Cooperative Oncology Group (ECOG) performance status of 0 or 1; (7) Adequate organ function confirmed within 7 days prior to treatment initiation; (8) For female patients, a negative pregnancy test result, no breastfeeding, and use of effective contraception for at least 3 months; for male patients, sterilization or effective contraception for at least 8 weeks; (9) Written informed consent and willingness to comply with study procedures.

#### Exclusion criteria

Patients meeting any of the following criteria will be excluded:

(1) History of tumor-related fistula formation; (2) High risk for gastrointestinal bleeding, esophageal fistula, or perforation; (3) Clinically significant poor nutritional status; (4) History of grade 3 or higher immune-related adverse events during definitive treatment, such as pneumonitis or myocarditis; (5) Active gastrointestinal disorders associated with an increased bleeding risk; (6) Symptoms or clinical signs suggestive of interstitial lung disease; (7) Severe or uncontrolled comorbidities; (8) History of another active malignancy within 2 years prior to enrollment; (9) Diagnosed autoimmune diseases or chronic use of systemic immunosuppressants or corticosteroids; (10) Inability to effectively communicate or adhere to required long-term follow-up; (11) Other conditions deemed unsuitable by the investigator.

Definitive treatment is defined as concurrent or sequential chemoradiotherapy delivered with curative intent, with a recommended radiation dose of at least 50.4 Gy in accordance with international guidelines. However, in patients who previously received immunotherapy in combination with chemoradiotherapy, dose de-escalation to a minimum of 45 Gy is permitted, taking into account individual tolerability and the potential synergistic effects of ICIs and radiotherapy, as determined by the treating physician.

### Randomization and allocation

Participants will be randomly assigned in a 2:1 ratio to the experimental or control group using simple randomization. Group allocation will be determined using computer-generated random numbers via an online randomization tool (https://www.randomizer.org/#randomize), which was applied uniformly to all enrolled participants. The randomization sequence is generated by an independent statistician and implemented through a password-protected online system, investigators obtain the assignment only after full eligibility is confirmed and have no access to the sequence or future allocations, thereby minimizing selection bias at enrolment.

### Neoantigen screening and synthesis

**Figure 3:**
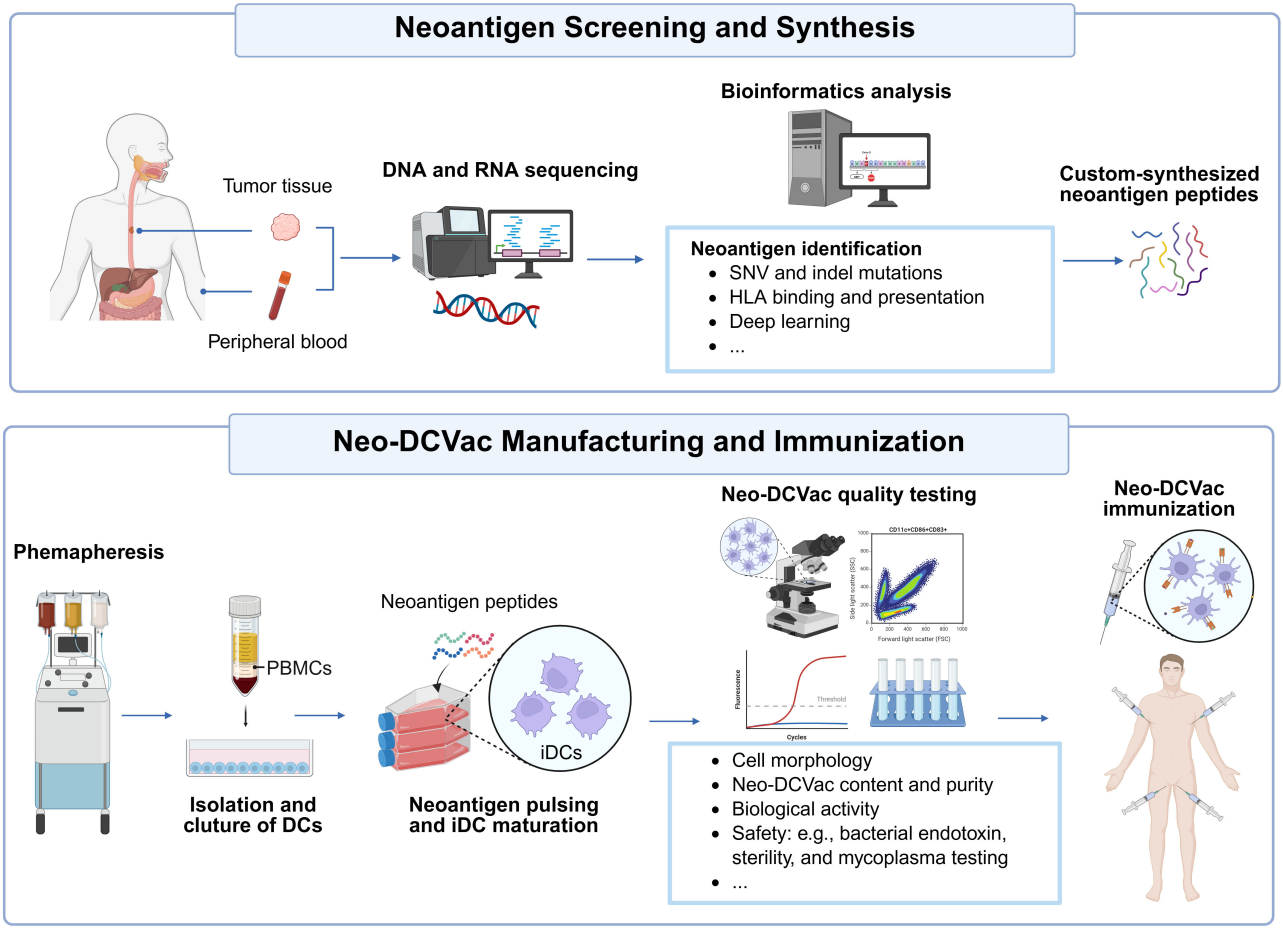
Overview of neoantigen selection and Neo-DCVac preparation. Following the collection of tumor tissue and peripheral blood samples, whole-exome sequencing (WES) and RNA sequencing (RNA-seq) are performed to identify somatic mutations. HLA typing is then conducted using sequencing data from both peripheral blood and tumor tissue, applying multiple computational tools. Candidate neoantigens are predicted based on peptide–HLA binding affinity and stability, HLA presentation scores, and immunogenicity assessments. After isolating leukocytes from peripheral blood through cell separation and centrifugation to obtain peripheral blood mononuclear cells (PBMCs), DCs are generated through *in vitro* culture. These DCs are subsequently co-cultured with chemically synthesized neoantigen peptides to induce maturation. Upon passing quality control evaluations, the personalized vaccine product, Neo-DCVac, is obtained and administered to patients via multiple subcutaneous injections targeting bilateral axillary and inguinal lymph nodes. SNV, single nucleotide variant; HLA, human leukocyte antigen; PBMCs, peripheral blood mononuclear cells; iDCs immature dendritic cells.

DNA and RNA sequencing will be performed on fresh tumor tissues or archived formalin-fixed paraffin-embedded (FFPE) pathology specimens collected within 1year. Sequencing will be conducted by Nanjing Geneseeq Technology Inc. Human leukocyte antigen (HLA) typing for class I (HLA-A, -B, -C) and class II (HLA-DR, -DQ, -DP) alleles will be performed using whole-exome sequencing data derived from patients’ peripheral blood mononuclear cells (PBMCs). Bioinformatic analyses will subsequently identify candidate neoantigens through multiple methods, including predictions of HLA-peptide binding affinity and stability, calculations of HLA presentation scores, and assessments of immunogenicity. The predefined selection criteria for neoantigen candidates include: (1) high predicted HLA-binding affinity (rank <0.5 or IC50 <50 nM); (2) significantly greater binding affinity of the mutant epitope compared with its wild-type counterpart; (3) high tumor variant allele fraction; (4) at least 5 supporting RNA-seq reads covering the mutation site; and (5) prioritization of mutations involving known oncogenes. Selected candidate neoantigen peptides will be synthesized by Shanghai Science Peptide Biological Technology Co., Ltd. (**Figure 3**).

### Vaccine manufacturing and immunization

Vaccine manufacturing is conducted at the GMP Facility of the Translational Medicine Infrastructure Management Department and the Center for Biotherapy, West China Hospital, Sichuan University (**Figure 3**). All procedures are performed under strict aseptic conditions. Peripheral blood mononuclear cells (PBMCs) will be isolated from patient leukocytes using a COM.TEC cell separator (Fresenius Kabi, Germany) at West China Hospital. Following the manufacturer’s instructions, approximately 100 mL of PBMCs are collected after 18–22 cycles of extracorporeal circulation. The harvested PBMCs were processed in a GMP-compliant lab. Monocyte-derived dendritic cells (DCs) will be generated from adhered PBMCs, as previously described (14). Briefly, PBMCs are cultured to differentiate into DCs, then co-incubated with personalized neoantigens peptides. Cytokines are added to induce DC maturation. On day 8, mature neoantigen-loaded DCs are harvested and pooled. Vaccine quality control includes assessment of cell viability (AIPO), morphology (Thermo microscope, USA), sterility (bacterial and mycoplasma testing by the Department of Laboratory Medicine at West China Hospital), and endotoxin levels (limulus amoebocyte lysate assay, Zhanjiang Andus Biotech Co., Ltd.). Phenotypic analysis via flow cytometry evaluates cell purity, uniformity, and expression of DC markers associated with maturation (CD83, CD86, CD11c), migration (CD209, CD11b), and chemotaxis (CCR7). The exact QC release criteria: minimum viable DC proportion ≥60%, total viable cell count ≥6×10^7^ cells per batch, negative sterility and mycoplasma tests, and endotoxin ≤5 EU/mL.

Neo-DCVac is prepared by suspending 0.5 to 2×10^7^ neoantigen-loaded DC in 2.5 mL of saline containing 1% human serum albumin. The vaccine must be administered within 2 hours after preparation and stored at 2–8 °C without freezing. Neo-DCVac is administered via multiple subcutaneous injections at bilateral axillary and inguinal lymph nodes (**Figure 3**).

The expected interval from tumor tissue acquisition to administration of the first Neo-DCVac dose is approximately 10–12 weeks, including about 2 weeks for vaccine manufacture and QC. During this period, patients continue camrelizumab maintenance and undergo regular radiologic monitoring. Before initiating vaccine manufacture, all patients undergo imaging, only those without disease progression by RECIST 1.1 proceed to vaccination and are included in the primary efficacy analysis. If progression is detected, Neo-DCVac may be offered on a compassionate-use basis. If a batch fails QC and sufficient material remains, manufacturing will be repeated. If not, the vaccine is discontinued and the patient continues standard care and follow-up.

### Study endpoints

The primary endpoint of the CHANT-241 trial is 2-year OS rate in patients receiving maintenance therapy with the Neo-DCVac plus camrelizumab. The Secondary endpoints include OS, progression-free survival (PFS) and treatment-related adverse events (TRAEs). Exploratory endpoints involve biomarker analyses, investigating associations between clinical outcomes and tumor mutational burden (TMB), programmed death-ligand 1 (PD-L1) expression, and circulating tumor DNA (ctDNA) dynamics. OS is defined as the duration from randomization until death from any cause, while PFS is defined as the interval from randomization to the first documented disease progression or death from any cause. TRAEs will be assessed using the National Cancer Institute Common Terminology Criteria for Adverse Events (NCI-CTCAE), version 5.0. These endpoints aim to identify potential predictive biomarkers of treatment response and long-term benefit.

### Treatment protocol

#### Experimental group

##### Bridging phase

Patients randomized to the experimental group will initially receive 2 to 3 cycles of camrelizumab administered every 4 weeks while awaiting preparation of the personalized Neo-DCVac.

##### Priming phase

The priming phase consists of 5 Neo-DCVac doses administered at weeks 1, 2, 4, 6, and 8. Cyclophosphamide (400 mg) will be intravenously administered one day prior to each Neo-DCVac injection. Additionally, granulocyte-macrophage colony-stimulating factor (GM-CSF; 0.075 mg) will be given subcutaneously on days 1 through 3 following each vaccination. During this phase, camrelizumab will continue to be administered every 4 weeks concurrently with Neo-DCVac.

##### Booster phase

Following the priming phase, Neo-DCVac combined with camrelizumab will continue to be administered every 4 weeks as maintenance therapy. Treatment will continue for up to 12 months or until disease progression, unacceptable toxicity, or death from any cause occurs.

Dose modifications of the Neo-DCVac are not permitted, as its efficacy and safety profile are not dose-dependent.

#### Control group

Patients in the control group will receive camrelizumab alone every 4 weeks, continuing for up to 12 months or until disease progression, unacceptable toxicity, or death from any cause occurs.

### Treatment outcome

#### Radiological assessment

Baseline imaging will be performed within 3 to 5 weeks after the completion of definitive therapy and prior to randomization. Evaluations will include contrast-enhanced computed tomography (CT) scans of the cervical, thoracic, and upper abdominal regions, as well as barium swallow radiography. Tumor response will be assessed using the Response Evaluation Criteria in Solid Tumors (RECIST), version 1.1. During the study, follow-up CT scans of the cervical, thoracic, and upper abdominal regions will be conducted every 12 weeks (± 1 week), regardless of any treatment delays or interruptions. Barium swallow radiography and esophagogastroduodenoscopy (EGD) are not mandatory but are recommended if patients develop relevant clinical symptoms. Imaging evaluations will be independently conducted by dedicated radiologists who are blinded to treatment allocation and will not be involved in clinical management.

#### Safety assessment

Safety monitoring involves continuous documentation and reporting of adverse events (AEs) and serious adverse events (SAEs), from patient enrollment through study completion, following regulatory and protocol-specific requirements. AEs occurring after informed consent but prior to study intervention initiation are also documented and reported. AE severity will be graded according to the National Cancer Institute Common Terminology Criteria for Adverse Events (NCI-CTCAE), version 5.0.

Clinical efficacy and safety evaluations will adhere to Good Clinical Practice (GCP) guidelines. Clinical research coordinators (CRCs) will record all data, which will be subsequently reviewed and verified by the principal investigator or designated sub-investigator.

### Follow Up

Long-term follow-up will be conducted throughout the clinical trial and after treatment completion. Survival status and long-term adverse events (AEs) will be continuously recorded until tumor recurrence, distant metastasis, patient death, or study closure, whichever occurs first.

### Data management

Study data will be collected using standardized case report forms (CRFs). All information will be derived from routine clinical records, including demographic data, vital signs, laboratory results, medication administration, and discharge status. Baseline characteristics and outcome measures will be documented for all randomized participants.

### Statistical analysis

All statistical analyses and graphs will be conducted using SPSS 25.0 (IBM Inc, Chicago, IL) or R 4.1.1 (https://www.r-project.org/). Kaplan-Meier method will be used to construct survival curves, including PFS and OS, and group comparisons were performed using the log-rank test. Rate ratios between groups were calculated with corresponding 95% confidence intervals (CIs) using the normal approximation method for large samples (n > 50) and the Newcombe–Wilson method for small sample sizes or proportions approaching 0 or 1. All statistical tests were two-sided, and a P value < 0.05 was considered statistically significant.

### Exploratory biomarker analysis

For exploratory biomarker analyses, ctDNA clearance at predetermined time points will be assessed, and its association with survival outcomes will be evaluated using Kaplan–Meier curves, log−rank tests, and Cox proportional hazards models. PD−L1 expression and tumor mutational burden (TMB) will be analyzed as continuous and categorical variables. Their prognostic and predictive values will be explored in Cox models, including treatment−biomarker interaction terms. Correlations among biomarkers (e.g., TMB, PD−L1, ctDNA) will be examined using Spearman’s rank correlation. All biomarker analyses are exploratory, without adjustment for multiplicity, and are intended to generate hypotheses for future studies.

## Discussion

Tumor immunotherapy has emerged as one of the most successful therapeutic strategies in oncology, primarily encompassing ICIs (33, 34), adoptive cell therapy (35, 36), and cancer vaccines (8, 37). Among these modalities, ICIs have become the standard of care for patients with advanced ESCC (20, 21, 38, 39). Cancer vaccines also demonstrate significant therapeutic potential by activating or enhancing host immune responses. Neoantigen-specific T cells, pivotal mediators of antitumor immunity, have been identified as ideal targets for cancer vaccines (5, 6). Personalized neoantigen-based immunotherapy can elicit tumor-specific T cell responses, thereby enhancing tumor sensitivity to ICIs (9, 40). Clinical studies have reported promising outcomes with neoantigen vaccines across various solid tumors (30, 41–44). However, these findings predominantly stem from early-phase trials, with definitive conclusions regarding their clinical efficacy limited by the absence of large-scale randomized controlled trials. Recent evidence from a 2024 randomized controlled trial demonstrated significant improvement in recurrence-free survival with a neoantigen vaccine combined with an ICI compared to ICI monotherapy in melanoma, thus confirming the clinical potential and manageable toxicity of neoantigen vaccines (25, 41, 43).

Despite these advancements, no standard maintenance therapy currently exists for patients with ESCC following definitive chemoradiotherapy. The PACIFIC trial, which demonstrated substantial survival benefits from maintenance immunotherapy in non–small cell lung cancer (NSCLC), has prompted consideration of analogous therapeutic paradigms for ESCC (45).

Neoantigen vaccines precisely target tumor-specific mutations, inducing robust T-cell responses, while ICIs reinvigorate exhausted T cells within the tumor microenvironment. Combining these therapies synergistically enhances the magnitude and persistence of antitumor immunity by coupling precise immune activation with sustained T-cell functionality (37, 46, 47). Our prior multicenter, single-arm study (NCT02956551) reported encouraging efficacy and favorable safety profiles for Neo-DCVac in patients with advanced NSCLC refractory to standard therapies, particularly when combined with ICIs (14). Additionally, compassionate-use data from postoperative adjuvant settings in locally advanced ESCC (CHANT-211 study) have also indicated synergistic efficacy between Neo-DCVac and ICIs.

The present trial builds upon this foundation and represents, to our knowledge, the first clinical investigation assessing Neo-DCVac as maintenance therapy following definitive chemoradiotherapy in patients with locally advanced, unresectable ESCC. This study is designed to systematically evaluate clinical outcomes and immune biomarkers, providing a comprehensive, multidimensional assessment of therapeutic efficacy. Additionally, planned mechanistic studies aim to elucidate the immunological basis underlying the clinical outcomes, offering essential insights to guide future optimization of neoantigen-based therapeutic strategies.

The individualized manufacturing process for Neo-DCVac introduces a meaningful risk of production delay or failure, which carries direct implications for the analytic strategy. We have therefore clarified the definitions of the intention-to-treat and per-protocol populations and outlined how any discrepancies between these analysis sets will be interpreted in the context of manufacturing feasibility. The use of a 2:1 randomization ratio further increases the operational demand on vaccine production; however, drawing on our prior multi-trial experience with neoantigen-based vaccines and the established capacity of our institutional GMP facility, this burden is considered acceptable for a single-centre phase II trial.

We also recognize that negative or modest clinical outcomes may reflect biological rather than operational limitations, including suboptimal neoantigen selection, insufficient vaccine-induced immunogenicity, or a profoundly immunosuppressive tumor microenvironment that is not adequately reversed by combination therapy with anti–PD-1. Consistent with the exploratory nature of this investigator-initiated trial, the study aims not only to evaluate preliminary efficacy signals but also to generate mechanistic insights. The planned translational analyses—encompassing neoantigen quality assessment, vaccine-induced immune responses, and comprehensive tumor-immune profiling—are designed to contextualize clinical outcomes and inform the rational refinement of future neoantigen-directed therapeutic strategies.

In addition to its potential strengths, this trial also faces several important operational and biological challenges. First, manufacturing failures or delays are an inherent risk of personalized vaccination and may influence the proportion of randomized patients who ultimately receive Neo-DCVac. All randomized patients will therefore be included in the intention-to-treat population, with complementary per-protocol and sensitivity analyses planned to assess the impact of manufacturing success or failure on outcomes. Second, the 2:1 randomization design increases the production burden. This is mitigated by conducting vaccine manufacture in a dedicated GMP Facility at the Center for Biotherapy, West China Hospital, Sichuan University, which operates under current GMP standards and contains more than 10 independent clean rooms, supported by a multidisciplinary team with extensive neoantigen-vaccine experience and a robust regional referral network to sustain accrual. Third, negative or modest efficacy results remain possible, for example due to suboptimal neoantigen selection, limited vaccine immunogenicity, or a profoundly immunosuppressive tumor microenvironment not fully reversed by Neo-DCVac plus camrelizumab. As an investigator-initiated phase II exploratory study, CHANT-241 is therefore designed not only to test efficacy signals but also to learn from failure, with predefined translational analyses (including neoantigen quality metrics, TMB/PD-L1/ctDNA, and immune profiling) planned to help interpret non-positive findings and optimize future Neo-DCVac combined with ICIs strategies.

## Conclusions

Currently, no studies have investigated personalized Neo-DCvac as a maintenance therapy in patients with unresectable, locally advanced ESCC. If effective, this strategy could offer a novel, personalized immunotherapeutic strategy for this population with limited post-treatment options.
